# Food Insecurity Determinants, Coping Strategies, and Association With Nutritional Status Among Hemodialysis Patients in Pahang, Malaysia: Protocol for a Mixed Methods Study

**DOI:** 10.2196/84575

**Published:** 2026-01-19

**Authors:** Nor Azwani Mohd Shukri, Sarah Muneera Karami, Wan Azdie Mohd Abu Bakar, Roselawati Mat Ya, Norhasmah Sulaiman

**Affiliations:** 1 Department of Nutrition Sciences Kulliyyah of Allied Health Sciences International Islamic University Malaysia Kuantan, Pahang Malaysia; 2 Food Security and Public Health Nutrition Research Group (FOSTER) Kulliyyah of Allied Health Sciences International Islamic University Malaysia Kuantan, Pahang Malaysia; 3 Department of Community Medicine Kulliyyah of Medicine International Islamic University Malaysia Kuantan, Pahang Malaysia; 4 Department of Nutrition Faculty of Medicine and Health Sciences Universiti Putra Malaysia Serdang, Selangor Malaysia

**Keywords:** coping strategies, food insecurity determinants, end-stage renal disease, food insecurity, hemodialysis, nutritional status

## Abstract

**Background:**

Food insecurity involves the lack of physical and economic access to sufficient, safe, and nutritious food that meets an individual’s dietary needs. Patients with end-stage renal disease (ESRD) undergoing hemodialysis have specific dietary needs, but food insecurity may hinder them from adhering to prescribed guidelines and preserving their nutritional status. However, no research has been conducted to elucidate food insecurity among patients undergoing hemodialysis in Malaysia.

**Objective:**

This study aimed to assess the prevalence of food insecurity, its determinants, and its association with nutritional status and explore the coping strategies used among patients undergoing hemodialysis in Pahang, Malaysia.

**Methods:**

This was a cross-sectional study that followed a mixed methods approach and was conducted with patients undergoing hemodialysis for ESRD at the Pahang Islamic Religious Council and Malay Customs (Majlis Ugama Islam dan Adat Resam Melayu Pahang [MUIP]) dialysis centers. The inclusion criteria were patients who were aged 18 years or older, were generally healthy, and had been undergoing hemodialysis regularly for at least 3 months. The food security status of the participants was determined using the Malay version of the Food Insecurity Experience Scale (M-FIES). The nutritional status included anthropometric measurements (height, weight, BMI, triceps skinfold [TSF] thickness, mid-upper arm circumference (MUAC), midarm muscle circumference (MAMC), and body fat percentage), biochemical parameters (serum urea, creatinine, albumin, phosphate, potassium, hemoglobin, and total iron-binding capacity [TIBC]), clinical assessments (Malnutrition Inflammation Score [MIS] and protein energy wasting [PEW]), and dietary intake (adherence to total calorie, protein, sodium, potassium, and phosphorus intake and diet monotony index [DMI]). The determinants were identified using logistic regression, while the association between food security status and nutritional status was analyzed using the chi-square or Fisher’s exact test and the independent *t* test. Semistructured interviews involved participants who were categorized as mildly, moderately, or severely food insecure in order to explore the contributing factors of food insecurity and how they coped with it based on their lived experience. The interviews were carried out until the data reached saturation, and then the data were analyzed thematically.

**Results:**

The study was funded by the Ministry of Higher Education Malaysia under the Fundamental Research Grant Scheme (FRGS/1/2023/SS10/UIAM/02/1) starting in September 2023. Data collection was conducted from December 2023 until August 2024, involving 287 participants, and data analysis has also been completed. As of January 2026, quantitative findings are under review and qualitative findings are being prepared.

**Conclusions:**

The implementation of this study protocol will provide new evidence to improve the understanding of food insecurity in this population. Elucidation of its key contributing factors, coping strategies, and potential connections to nutritional status in this population can help guide more informed policymaking and effective interventions.

**International Registered Report Identifier (IRRID):**

RR1-10.2196/84575

## Introduction

The burden of food insecurity continues to rise worldwide, impacting individuals in diverse socioeconomic and geographic settings. Foor insecurity is increasingly recognized as a growing public health concern in both high- and middle-income countries. The 1996 World Food Summit defined food insecurity as a condition in which individuals lack physical, social, and economic access to sufficient, safe, and nutritious food that meets their dietary needs and food preferences for an active and healthy life [1]. It is associated with cardiometabolic risks, such as obesity, diabetes, hypertension, dyslipidemia, and stress [2]. According to the *2025 Global Report on Food Crises* (GRFC), acute hunger has risen for the sixth consecutive year, with 295.3 million people across 53 countries in the world experiencing acute food insecurity in 2024—threefold the number in 2016 and double that in 2020 [3].

Food insecurity can result in poor nutrition, an elevated risk of chronic diseases, and a deterioration of the health outcomes of patients with chronic kidney disease (CKD), as well as contributing toward the progression of CKD to end-stage renal disease (ESRD) [4,5]. This study is informed by Campbell’s conceptual framework [6], which hypothesizes food insecurity as multidimensional, influenced by the researcher’s conceptualization of its social context, such as socioeconomic characteristics, food access, and health-related factors, and linked to downstream nutritional and clinical outcomes. Previous studies among patients with CKD have identified demographic, socioeconomic, and social factors, including age, marital status, income, educational level, employment status, financial constraints, limited access to transportation, lack of social support, and a high financial burden, as key determinants of food insecurity [7-9].

Food insecurity has been reported to affect 36% [7] and 26.3% [10] of patients undergoing hemodialysis and is a critical issue among these patients since they have special dietary needs for preserving health. Adequate calorie and protein intake and restricted sodium, potassium, and phosphorus intake, with controlled fluid, are required to prevent malnutrition [11]. Patients with CKD facing food insecurity report greater difficulties in adhering to a costly renal diet compared to those without food insecurity [10]. Exacerbated by financial difficulties and inadequate transportation, which are associated with restricted access to nutritious food, the presence of food insecurity may pose challenges for patients undergoing hemodialysis to comply with these dietary guidelines and maintain their nutritional status [9].

Coping mechanisms are crucial for individuals or households experiencing food insecurity to facilitate managing limited resources and mitigating the immediate impact of inadequate food or resources to buy food. Individuals with chronic conditions (eg, diabetes, HIV/AIDS, and cancer) and food insecurity have adopted coping strategies that fall into two categories: food related and nonfood related. These strategies include consuming less preferred food [12], reducing the meal frequency [12], skipping meals [13], prioritizing food expenses over medical treatments [13], relying on low-cost food to save money for medical supplies [14], and receiving food assistance [13,15].

Although food insecurity among patients with CKD has been widely studied in high-income countries, such as the United States [7,9,10], evidence from Southeast Asia, including Malaysia, remains limited. Malaysia presents a unique context due to its mixed public-private health care system, the rising cost of living, and the increasing prevalence of CKD requiring long-term hemodialysis. Understanding food insecurity within this local context is essential, as socioeconomic conditions, food accessibility, and health care–financing systems may vary substantially from those reported from other regions.

Given the limited local evidence and the unique clinical and socioeconomic challenges faced by patients undergoing hemodialysis in Malaysia, this mixed methods study aims to examine food insecurity among patients undergoing hemodialysis using a quantitative approach and to explore the phenomenon in depth through qualitative inquiry based on the lived experiences of these patients. Specifically, the study aims to (1) assess the prevalence of food insecurity, (2) identify its determinants, (3) examine the association between food security status and nutritional status, and (4) explore the contributing factors and coping strategies related to food insecurity among patients undergoing hemodialysis in Pahang, Malaysia.

## Methods

### Study Design

This cross-sectional study used a mixed methods approach, which includes quantitative and qualitative data collection. This approach was followed to complement and expand the findings from the quantitative research by seeking elaboration and clarification through qualitative research. Data collection was conducted sequentially. It commenced with a broad survey involving a large number of people to generalize the findings to a population, followed by a second phase that included detailed qualitative, semistructured interviews for a deeper exploration of the experiences of selected cases or individuals with food insecurity [16]. The timing, setting, and sequence of data collection activities for both the quantitative and qualitative phases are summarized in Table 1.

**Table 1:** Overview of study procedures, measures, and data collection schedule.

Data collection activity and variables/measures		Timing	Setting
**Quantitative questionnaire**			
	Food security status	During dialysis sessions	Dialysis center
	Sociodemographic and other risk factors of food insecurity	During dialysis sessions	Dialysis center
**Quantitative nutritional status assessments**			
	Anthropometric measurements	During and after dialysis sessions	Dialysis center
	Biochemical parameters	From medical records	Dialysis center
	Clinical status assessments	During dialysis sessions	Dialysis center
	Dietary intake assessments	During dialysis sessions	Dialysis center
**Qualitative semistructured interviews**			
	Contributing factors and coping strategies related to food insecurity	Outside dialysis hours, scheduled separately	Telephone call

### Study Population

This study was conducted with patients undergoing hemodialysis at four dialysis centers managed by the Pahang Islamic Religious Council and Malay Customs (*Majlis Ugama Islam dan Adat Resam Melayu Pahang* [MUIP]). The council conducts social development programs as part of its *zakat* administration, which is an Islamic charitable contribution aimed at supporting those in need, through the establishment of centers that provide hemodialysis services to patients with ESRD who have a low socioeconomic status or who are classified as *asnaf* (individuals deemed eligible to receive *zakat* funds). Each of these dialysis centers is in the Kuantan, Gambang, Pekan, and Pusat Bandar Jengka areas, covering three different districts in Pahang, one of the east coast states of Malaysia.

### Ethical Considerations

This study was approved by the International Islamic University Malaysia Research Ethics Committee (ID IREC 2024-001). Administrative permission from the MUIP dialysis centers was obtained before data collection. Before the study commenced, its details were explained to all participants, and written informed consent was obtained from them. Their personal information was anonymized, and confidentiality was maintained throughout the study.

### Quantitative Phase

#### Research Framework

**Figure 1:**
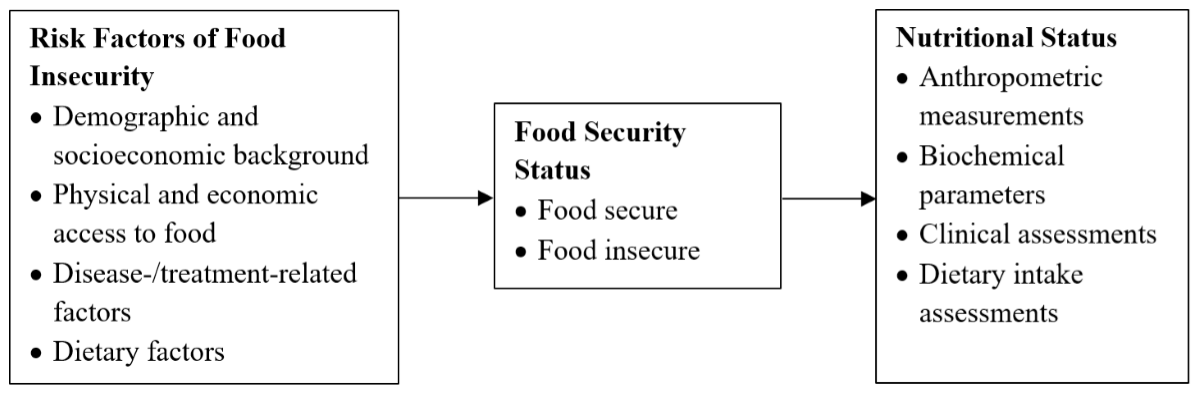
Research framework of the quantitative phase of the study.

The quantitative phase of this study was guided by a research framework that focuses on the determinants of food insecurity among patients undergoing hemodialysis and the association of food insecurity with nutritional status (Figure 1). Based on previous studies, demographic and socioeconomic data [8,17,18] as well as physical and economic access to food [19,20] are the known determinants of food security status. However, since this study was conducted with patients undergoing hemodialysis, disease-/treatment-related factors [8,14,21,22] could be one of the variables affecting their food security status. Nutrition literacy and dietary adherence levels of participants [23] were also assessed as potential risk factors to investigate whether the study population is experiencing food insecurity due to a lack of knowledge about a healthy diet for hemodialysis or compliance with dietary recommendations. Furthermore, Campbell’s conceptual framework of risk factors and consequences of food insecurity [6] depicts food insecurity as having an impact on nutritional status.

#### Participant Recruitment and Sampling

The inclusion criteria for the study were as follows:

- Age at least 18 years
- Undergoing regular hemodialysis for 4 hours per session, three times a week
- Undergoing dialysis for at least 3 months before enrolling in the study
- No major acute diseases or psychological disorders

The exclusion criteria were as follows:

- Inability to hear, understand, or speak well
- Not mentally and physically fit to complete all the assessments

All patients undergoing hemodialysis who fulfilled the inclusion criteria were included in this study, except those who were unresponsive after being approached at least three times. Universal sampling was applied in the quantitative phase, whereby all eligible patients undergoing hemodialysis at the MUIP dialysis centers during the study period were invited to participate. As this approach represents a census of the accessible population, a fixed sample size was not predetermined. Instead, the precision of the prevalence estimate was evaluated using the margin of error (d) derived from a single-proportion formula. Assuming a food insecurity prevalence of 26.3% among patients undergoing hemodialysis, as reported previously [9], a sample size of approximately 250 participants would correspond to a margin of error of 5% at a 95% confidence level. Recruitment beyond this number was considered sufficient to ensure adequate precision of the study estimates.

#### Data Collection and Instruments

Data collection was conducted in the Malay language using interviewer-administered questionnaires, during which the researcher asked the participants questions and recorded their responses.

##### Objective 1: Assessing the Prevalence of Food Insecurity Among Patients Undergoing Hemodialysis

The food security status of participants was ascertained using the Malay version of the Food Insecurity Experience Scale (M-FIES) [24], which consists of eight questions that assess the severity of food insecurity at the household or individual level, as determined by the individuals’ self-reported behaviors and experiences with respect to one of the dimensions of food insecurity, food accessibility [1]. Participants were required to respond with either “yes” or “no” to each question. An affirmative response was assigned a score of 1, while a negative response was assigned a score of 0. The sum of scores from the eight questions was further categorized into the following severity levels: food security (0), mild food insecurity (1-3), moderate food insecurity (4-6), and severe food insecurity (7-8). For data analysis, the M-FIES categories of mild, moderate, and severe food insecurity were combined and classified as “food insecurity.”

##### Objective 2: Identifying the Determinants of Food Insecurity Among Patients Undergoing Hemodialysis

To identify the determinants of food insecurity, four main risk factors of food insecurity were included in the questionnaire. For demographic and socioeconomic data, questions about the participants’ age, gender, ethnicity, marital status, educational level, employment status, total monthly income, financial assistance status, living area, and social support status were asked. Social support status was determined using the adapted Malay version of the Multidimensional Perceived Social Support Scale (MSPSS-M) [25,26]. Only 8 of 12 questions to measure an individual’s perception of support from family and friends were included. To calculate the mean subscale score for the family subscale, the sum of scores for items 1, 2, 5, and 7 was divided by 4, while for the friend subscale, the sum of scores for items 3, 4, 6, and 8 was divided by 4. A mean scale score between 1 and 2.9 was classified as low social support, a score between 3 and 5 was classified as moderate social support, and a score between 5.1 and 7 was classified as having high social support.

Questions regarding physical and economic access to food included shop availability, the distance from the house, food variety, the affordability of purchasing food, transport ownership, food delivery status, and weekly food expenditure. This section also inquired about the disease- and treatment-related data of participants, such as the presence of comorbidities, duration of the disease, dialysis vintage, and self-reported appetite rating. For the dietary part, two validated questionnaires, the Malay version of the Dialysis-Specific Nutrition Literacy Scale (DSNLS) [27] and the dietary adherence component of the modified End-Stage Renal Disease Adherence Questionnaire (ESRD-AQ) [28], were used to assess the participants’ nutrition literacy and dietary adherence, respectively.

##### Objective 3: Determining the Association Between Food Security Status and Nutritional Status of Patients Undergoing Hemodialysis

To determine how food security status affects the nutritional status of patients undergoing hemodialysis, this component of the study assessed participants’ anthropometric measurements, biochemical parameters, clinical characteristics, and dietary intake.

#### Anthropometric Measurements

The participants’ height, (postdialysis) dry weight, BMI, triceps skinfold (TSF) thickness, mid-upper arm circumference (MUAC), midarm muscle circumference (MAMC), and total fat percentage were measured. The anthropometry status assessment was only conducted after the participants completed the hemodialysis session.

##### Height

The participants’ height was measured using a stadiometer placed upright against a straight wall surface to ensure accurate measurement. The procedure was as follows:

- The participant was requested to remove their shoes and hat, if any.
- The head plate of stadiometer was raised to make enough space for the participant to stand underneath it.
- The participant was instructed to step onto the stadiometer; stand upright against the rod, with their heels together and arms on the face outward; and face forward.
- The participant’s head was ensured to be in Frankfort position.
- The headboard was lowered onto the highest point of the participant’s head with sufficient pressure to compress the hair.
- The height was read at eye level and recorded to the nearest 0.5 cm. The height was measured three times, and the median value was recorded as the final measurement.

For participants who were unable to stand, height was measured by knee height as follows:

- The participant was seated comfortably with feet flat on the floor and knees positioned at a 90° angle.
- Any footwear was removed to ensure accuracy, and a measuring tape was used for measurement.
- The participant’s thigh was positioned parallel to the floor.
- The measuring tape was positioned with its upper end placed on the top of the participant’s knee.
- The knee height was measured from the bottom of the heel (or the sole if the heel was not accessible) to the tope edge of the measuring tape.
- The measurement was recorded to the nearest centimeter.
- The same process was repeated on the other leg to ensure consistency, and the average of the two readings was used, if appropriate.

##### Postdialysis Dry Weight

A digital weighing scale (Seca 677) was used to measure the postdialysis dry weight of the participants as follows:

- The participant was asked to remove their shoes and all objects that could affect weight measurement, if any.
- The participant was instructed to step onto the scale and face forward.
- The weight was measured three times, and the median value was recorded as the final measurement.

##### Body Mass Index

The participants’ BMI was calculated as weight (kg)/height squared (m^2^). The value was interpreted using the BMI classification [29] shown in Table 2.

**Table 2:** BMI classification.

BMI range (kg/m²)	Classification
<18.5	Underweight
18.5-24.9	Normal
25.0-29.9	Overweight
≥30.0	Obese

##### Triceps Skinfold Thickness

The participants’ TSF thickness was measured on the nonfistula arm using the Harpenden skinfold caliper (HSK-BI, British Indicators) as follows:

- The acromion and olecranon processes were marked. A flexible, nonstretchable measuring tape (Crescent Lufkin W606PD, Apex Tool Group) was used to mark the midpoint between the two processes on the lateral aspect of the arm.
- The measuring tape was wrapped around the arm horizontally.
- The midpoint mark was drawn on the back of the arm to indicate the location for positioning the caliper.
- The caliper was placed 1 cm below the finger. The TSF was measured three times, and the median value was recorded as the final measurement.

##### Mid-Upper Arm Circumference

The participants’ MUAC was measured using a flexible, nonstretchable measuring tape (Crescent Lufkin W606PD, Apex Tool Group). The measurement was obtained at the midpoint of the upper arm between the acromion and olecranon processes.

##### Midarm Muscle Circumference

Subsequently, the participants’ MAMC was calculated using the following formula:

MAMC (cm) = MUAC (cm) – [TSF (cm) × π]

The value was interpreted according to the MAMC Standard Reference [30] shown in Table 3.

**Table 3:** MAMC^a^ standard reference.

Gender	Standard reference (cm)	90% of standard reference: moderately malnourished (cm)	60% of standard reference: severely malnourished (cm)
Men	25.3	22.8	15.2
Women	23.5	20.9	13.9

^a^MAMC: midarm muscle circumference.

##### Body Fat Percentage

The participants’ fat mass was measured using a body composition analyzer (Omron Body Composition Monitor HBF-702T) according to the manufacturer’s guidelines [31] and interpreted as per Table 4. The procedure was as follows:

- The participant was asked to remove their stockings.
- The participant was instructed to step onto the body composition analyzer and lift the handle of the instrument using both hands.
- The value displayed on the monitor was recorded.

**Table 4:** Healthy body fat percentage by age.

Age range (years)	Body fat in men (%)	Body fat in women (%)
20-39	8-19	21-32
40-59	11-21	23-33
60-79	13-24	24-35

#### Biochemical Parameters

Serum urea, creatinine, albumin, phosphate, potassium, hemoglobin, and total iron-binding capacity (TIBC) values were retrieved from the patients’ medical records of routine three-monthly blood assessments. The status of each parameter (normal vs abnormal) was classified based on the reference range provided in the laboratory reports.

#### Clinical Assessments

### Malnutrition Inflammation Score

The participants’ Malnutrition Inflammation Score (MIS) was determined during anthropometric assessment. The tool is divided into four sections: (12) nutritional history (change in dry weight, dietary intake, gastrointestinal symptoms, functional capacity, and comorbidities with dialysis vintage), (2) physical examination (decrease in fat stores or loss of subcutaneous fat and signs of muscle wasting), (3) BMI, and (4) laboratory values (serum albumin and TIBC). Each component has four severity levels, scored from 0 (normal) to 1 (mild), 2 (moderate), and 3 (very severe). The sum of all 10 MIS components ranges from 0 to 30, with a higher score reflecting a more severe degree of malnutrition and inflammation [32]. Scores ≥8 indicated malnourishment, while scores <8 indicated that participants were well-nourished, as validated in a local study [33].

### Protein Energy Wasting

Protein energy wasting (PEW) indicates a state of decreased protein and energy stores and is associated with reduced functional capacity, impaired quality of life, and increased morbidity and mortality in patients with CKD [34]. The diagnostic criteria proposed by the International Society of Renal Nutrition and Metabolism Expert Group [34] was adopted to identify the presence of PEW among the participants. The PEW diagnosis comprises four main criteria: biochemical assessment; low body weight, reduced total body fat, or weight loss; decreased muscle mass; and low energy or protein intake. A diagnosis of PEW was made if any three of the four criteria were met: serum albumin<3.8 g/dL, BMI<23 kg/m^2^, >10% reduction in MAMC in relation to the 50th percentile of a reference population, and daily dietary energy intake<25 kcal/kg ideal body weight (IBW) [35].

#### Dietary Intake Assessments

A multiple-pass 24-hour diet recall technique [36] was used to evaluate participants’ dietary intake of total energy, protein, and micronutrients, such as sodium, potassium, and phosphorus, covering 3 days (one dialysis day, one nondialysis day, and one weekend day). Face-to-face interviews were conducted by a trained dietitian (the researcher). The dietary data were analyzed using Nutritionist Pro diet analysis software with the USDA Food Database and Malaysian Food Composition Tables (Axxya System LLC). Each participant’s usual dietary intake was calculated from the average of the three 24-hour dietary recalls. The residual method was used to adjust protein intake for total energy intake to reduce the effect of measurement errors, such as misreporting of energy intake, which could potentially impact the overall nutritional values [37]. The protein intake that corresponded with the mean total energy intake of the study population was adjusted by adding the residual (the difference between observed nutrient values for each participant and values predicted by the regression equation) to the intake. As such, protein intake was adjusted for total energy intake using the residual method calculated from regression, with total energy serving as the independent variable and protein intake as the dependent variable. Therefore, the residuals were then used as energy-adjusted protein intake in further analyses.

The adequacy of energy and protein intake was determined through comparison with the Kidney Disease Outcomes Quality Initiative (KDOQI) clinical practice guidelines for nutrition in chronic renal failure [10]. The daily recommended energy intake for patients undergoing hemodialysis is 30-35 kcal/kg IBW, while the recommended protein intake is 1.0-1.2 g/kg IBW per day. The patient’s body weight is determined using the dry weight that is taken following the completion of the dialysis session. For micronutrient intake, patients with CKD who are undergoing dialysis are advised to limit their sodium intake to less than 2300 mg/day [10,38]. Their daily potassium intake should not surpass 2730 mg/day [38]. It is also recommended that the daily intake of phosphorus be less than 1000 mg/day [10,38]. Participants who adhered to the recommended dietary intake values were classified as “adhere,” while those who exceeded the values were classified as “not adhere.”

Dietary intake assessment also included the evaluation of dietary variety using the diet monotony index (DMI), which was calculated based on the serving sizes of 27 food groups, as assessed through the 24-hour dietary recall [39,40]. The proportion of total servings per week accounted for by each specific food was quantified. The DMI was calculated as follows:

[(P_1_)^2^ + (P_2_)^2^ + (P_3_)^2^ + . . . + (P_n_)^2^] × 100,

where P_1_, P_2_, . . ., P_n_ are the proportion of total servings from each specific food. The resulting score mainly reflected food groups that represent the majority of total consumption. Consequently, higher DMI scores indicated a more monotonous diet, while lower scores suggested a greater variety of diets [39].

#### Statistical Analysis

IBM SPSS was used to conduct statistical analyses. Descriptive statistics was used to explore the participants’ demographic and socioeconomic characteristics, physical and economic access to food, disease-/treatment-related factors, dietary factors, and food security status. Bivariate logistic regression was used to identify the association between each possible determinant and food security status. Variables with *P*<.25 in the bivariate logistic regression were included in the multivariable logistic regression model to determine independent associations with adjusted odds ratios (aORs) [41,42]. The area under the receiver operating characteristic (ROC) curve (AUC) was also tested on the regression model to assess model discrimination [43]. The values ranged from 0 to 1, where 0.5 indicated that the model is useless for discrimination, while values near 1 indicated that the model has a high predictive power and can discriminate between the two outcome groups. The statistical tests that were used to determine the association between food security status and nutritional status are presented in Table 5. The statistical significance value was set at *P*<.05.

**Table 5:** Statistical tests to determine the association between food security status (independent variable) and nutritional status.

Dependent variables		Statistical test
**Anthropometric measurements**		
	Height	Independent *t* test
	Weight	Independent *t* test
	BMI status	Chi-square test (or Fisher’s exact test)
	TSF^a^	Independent *t* test
	MUAC^b^	Independent *t* test
	MAMC^c^	Independent *t* test
	Total fat	Independent *t* test
**Biochemical parameters**		
	Serum urea	Chi-square test (or Fisher’s exact test)
	Serum creatinine	Chi-square test (or Fisher’s exact test)
	Serum albumin	Chi-square test (or Fisher’s exact test)
	Serum phosphate	Chi-square test (or Fisher’s exact test)
	Serum potassium	Chi-square test (or Fisher’s exact test)
	Hemoglobin	Chi-square test (or Fisher’s exact test)
	TIBC^d^	Chi-square test (or Fisher’s exact test)
**Clinical assessments**		
	MIS^e^	Chi-square test (or Fisher’s exact test)
	PEW^f^	Chi-square test (or Fisher’s exact test)
**Dietary intake assessments**		
	Adherence to total calorie intake	Chi-square test (or Fisher’s exact test)
	Adherence to protein intake	Chi-square test (or Fisher’s exact test)
	Adherence to sodium intake	Chi-square test (or Fisher’s exact test)
	Adherence to potassium intake	Chi-square test (or Fisher’s exact test)
	Adherence to phosphate intake	Chi-square test (or Fisher’s exact test)
	DMI^g^	Independent *t* test

^a^TSF: triceps skinfold.

^b^MUAC: mid-upper arm circumference.

^c^MAMC: midarm muscle circumference.

^d^TIBC: total iron-binding capacity.

^e^MIS: Malnutrition Inflammation Score.

^f^PEW: protein energy wasting.

^g^DMI: diet monotony index.

### Qualitative Phase

#### Participant Recruitment and Sampling

Participants for the qualitative component of this study included patients undergoing hemodialysis with different levels of food insecurity. They were a subgroup of the main study population. Upon completion of the quantitative phase of the study, some of the participants deemed suitable for interviews were approached by the researcher. Using purposive sampling, those who were classified as having mild, moderate, or severe food insecurity based on their M-FIES score were invited to participate in semistructured interviews. They were purposively selected to achieve approximately equal representation across the four hemodialysis centers and the three categories of food insecurity. The interview’s purpose and process were explained to them, and if they agreed, their phone numbers were recorded.

#### Data Collection and Instruments

##### Objective 4: Exploring the Contributing Factors and Coping Strategies Related to Food Insecurity Among Patients Undergoing Hemodialysis

A semistructured interview guide adopted and adapted by a previous study [44] was used to assist the interviewer and to maintain consistency during the interview session. The guidelines include a brief welcome of the interviewee, an explanation about the procedure, and questions and prompts related to the topic (Multimedia Appendix 1). Before conducting the interview, the researcher familiarized themselves with the interview guide to ensure a smooth session. A pilot test was also conducted to ascertain the anticipated duration of the session.

A one-to-one, semistructured in-depth interview format was used since this study aims to investigate food insecurity, which is considered a sensitive issue because it is closely related to the fundamental aspects of health and well-being and carries emotional and social consequences. Additionally, a semistructured interview is useful in a mixed methods study, as it discovers participants’ motivations, attitudes, beliefs, and the impact of specific events in their lives, in addition to supplementing and adding depth to the quantitative approach [45,46].

Prior to the interview session, the participants were approached by the researcher via WhatsApp. A message was sent to each participant, outlining the interview process and the expected duration of the session. If they agreed, the researcher inquired about their preferred date and time for the interview. A one-to-one interview session was subsequently conducted over the phone. In the case of unanswered messages, the researcher made three attempts to reach the participant over the phone during working days. If they were still unreachable, the participant was excluded from the interview list. The interview did not take long, as the guide contained only two main questions that needed to be answered by the participants. To ensure consistency and comparability of data collection, all interviews were conducted over the phone.

Before the interview began, consent from each participant was sought to audio-record the session using a digital voice recorder (TX660, Sony Corporation) as the main recorder and an audio-recording app (macOS, Apple Inc) as a backup recorder. Participants were informed that they could refuse to answer questions and withdraw from the study at any time. The interviews were conducted in Malay until data saturation was achieved, indicating the point when no new information was obtained from the participants and when subsequent interview transcripts yielded no new codes or themes [47]. This was assessed through ongoing comparative analysis using NVivo software, where redundancy in codes and conceptual categories was observed across interviews. Probing techniques, such as repeating the interviewee’s statements, summarizing the main idea, and demonstrating engagement through verbal agreement, were used to encourage participants to persist in the conversation, enabling them to provide additional information that could be useful for understanding the topic [46,48]. In addition, the key points and main ideas highlighted during the interviews were noted to guide follow-up questions, ensure that all interview questions were answered, and assist in transcription [45].

#### Qualitative Analysis

Assisted by the field notes, a word-by-word, verbatim transcription of the audio-recorded interviews was manually composed by the researcher, while ensuring an accurate and detailed record of spoken dialogue. To ensure that the recorded audio was accurately transcribed, the researcher listened to it multiple times during the transcription process. Thematic analysis (systematically identifying, organizing, and delivering information about patterns of meaning [themes] in a dataset [49]) of the qualitative data was performed using NVivo software.

Trustworthiness was ensured during each phase of thematic analysis through several methods [50]. Credibility was enhanced through familiarity with data. The researcher familiarized themselves and thoroughly engaged with the data to ensure comprehension, given the data’s extensive range and profound nature. Thus, before beginning coding, the complete dataset was thoroughly reviewed; this process may influence the development of ideas and the identification of potential patterns as researchers gain a comprehensive understanding of the data [51]. In addition, transferability was ensured through the inclusion of direct participant quotations, which will provide conceptual depth and allow readers to access the applicability of the findings to other settings. Initial codes or subthemes from the potentially relevant data in the transcript were generated, which will enable the researcher to simplify and concentrate on particular characteristics of the data.

An inductive approach was followed in defining themes and subthemes, where the codes arose from the data themselves and did not rely on any existing theories or frameworks [52]. Dependability was applied through iterative coding, where the development of codes continued; eventually, similar groups of coded data extracts were sorted into several main themes. The coded data extracts for each theme were then reviewed to determine whether they exhibited a cohesive pattern. Lastly, these themes were labeled and defined, and their significance and rationale were determined [51]. Confirmability was supported by refining themes to ensure they were grounded in the participants’ narratives. To enhance analytical rigor, initial coding and theme development were independently reviewed by other members of the research team, and any discrepancies were discussed until consensus was achieved. Following the thematic analysis, the tabulated themes and supporting quotes were clarified with the research team to ensure accuracy, consistency, and credibility of the findings.

## Results

The study was funded by the Ministry of Higher Education Malaysia under the Fundamental Research Grant Scheme (FRGS/1/2023/SS10/UIAM/02/1) starting in September 2023., involving 287 participants, and data analysis has also been completed. As of January 2026, quantitative findings are under review and qualitative findings in preparation. A total of 287 participants were enrolled, which exceeded the minimum number required to achieve the targeted margin of error. For the quantitative phase of the study, data collection was conducted from December 2023 until August 2024. For the qualitative phase, 19 (6.6%) participants, drawn from the main study population (N=287), participated in the semistructured interviews. Data analysis was also completed. The manuscript reporting findings from the quantitative phase (prevalence and determinants of food security status and its association with dietary intake) was submitted for publication in December 2025 and is currently under review. Meanwhile, the manuscript reporting findings from the qualitative phase (contributing factors and coping strategies related to food insecurity) is currently being prepared for publication.

In addition, two questionnaires, the Malay version of the DSNLS [27] and the dietary adherence component of the modified ESRD-AQ [28], were translated and validated in this study to assess the participants’ nutrition literacy level and dietary adherence, respectively.

## Discussion

### Summary

This study is expected to provide insight into the prevalence of food insecurity among patients undergoing hemodialysis in Pahang, Malaysia, and to examine the hypothesized determinants of food insecurity, related to demographic and socioeconomic characteristics, physical and economic access to food, disease-/treatment-related factors, and dietary factors. Specifically, the study will evaluate the relationship between food security status and nutritional status indicators, including anthropometric measurements, biochemical parameters, clinical assessments, and dietary intake. The qualitative component will further explore, through semistructured interviews, contributing factors and coping strategies used by patients with food insecurity undergoing hemodialysis.

### Strengths and Limitations

The main strength of this study is that it is the first to assess food insecurity among patients undergoing hemodialysis in Malaysia. This study is also the first to explore coping strategies for food insecurity among patients undergoing hemodialysis. In terms of the study design, it used a mixed methods approach that ensures richness of data and provides a comprehensive understanding of data. The findings from the semistructured in-depth interviews are expected to complement the quantitative component of the study by adding nuance to the questionnaire data, providing a more profound understanding of the experiences of patients with food insecurity undergoing hemodialysis and thus strengthening the knowledge about this issue. Another strength is the use of a universal sampling method that eliminated sampling bias by including all patients undergoing hemodialysis at the selected dialysis centers who met the inclusion criteria, ensuring complete coverage of the population. However, it may not be generalizable to other settings and may lack external validity since the characteristics of the group being studied may not represent patients undergoing hemodialysis in other settings.

However, the protocol has a few limitations. Several variables included in the questionnaires were based on participants’ self-reports, such as the duration of disease, dialysis vintage, frequency of dietary education, and adherence to diet, as well as dietary assessments, which require participants to recall the food consumed in the past 24 hours and the past few days (including weekends and the nondialysis day). This method may introduce recall bias if the participants do not accurately remember their past food intake. Over- or underestimation of food intake may also occur, particularly in participants’ responses, either unintentionally or to seem compliant with dietary recommendations. However, the effect of reporting inaccuracies was minimized by using a statistical adjustment method that adjusted protein intake based on total energy intake, thereby reducing measurement errors. Additionally, using phone call interviews may have overlooked the participants’ nonverbal communications, including body language, gestures, and facial expressions. This method would therefore limit additional information useful for the interviews. Hence, face-to-face settings may be advantageous for implementation in further research to improve participants’ attention and offer more detailed responses during the interviews.

### Conclusion

This protocol outlines a mixed methods study that will generate new evidence and valuable resources for understanding the prevalence and determinants of food insecurity among patients undergoing hemodialysis, its association with nutritional status, and the coping strategies used employed by individuals with food insecurity. The qualitative findings are anticipated to provide useful complementary insights derived from the participants’ lived experiences, in addition to the quantitative data obtained through statistical analysis. Collectively, these findings are expected to inform clinical management by supporting early identification of nutritionally vulnerable patients undergoing hemodialysis and guiding targeted nutrition interventions. In addition, evidence generated may support policy-level initiatives aimed at integrating food security screening and assistance into renal care services. By addressing food insecurity through both clinical and policy perspectives, this study has the potential to contribute to improved nutritional care, health outcomes, and quality of life of patients undergoing hemodialysis.

## Appendix

Multimedia Appendix 1 Interview guidelines.

Multimedia Appendix 2 Peer-review report from the Fundamental Research Grant Scheme (FRGS), Ministry of Higher Education, Malaysia.

## Abbreviations

CKD: chronic kidney disease
DMI: diet monotony index
DSNLS: Dialysis-Specific Nutrition Literacy Scale
ESRD: end-stage renal disease
ESRD-AQ: End-Stage Renal Disease Adherence Questionnaire
IBW: ideal body weight
MAMC: midarm muscle circumference
M-FIES: Malay Food Insecurity Experience Scale
MIS: Malnutrition Inflammation Score
MUAC: mid-upper arm circumference
MUIP: Majlis Ugama Islam dan Adat Resam Melayu Pahang
PEW: protein energy wasting
TIBC: total iron-binding capacity
TSF: triceps skinfold

## Data Availability

The datasets generated and analyzed during this study are available from the corresponding author (NAMS) upon reasonable request.
