# Supplementary material for: Food Insecurity Determinants, Coping Strategies, and Association With Nutritional Status Among Hemodialysis Patients in Pahang, Malaysia: Protocol for a Mixed Methods Study
Source: JMIR Res Protoc. 2026 Jan 19;15:e84575. doi: 10.2196/84575 (PMC12904101; doi:10.2196/84575)
Supplement: Multimedia Appendix 1 [file resprot_v15i1e84575_app1.pdf]

Interview Guideline

1. A brief welcome to the interviewee.
2. Explain to the interviewee:
   - The procedure and structure of the interview
   - The use of audio recording
   - The interviewee's right to withdraw from participation at any time
3. Start the recorder.
4. Conduct the interview.
5. Thank the interviewee for their participation and stop the recorder.

<u>Script (in Malay)</u>

a. *Pertama sekali, terima kasih kerana sudi untuk menyertai temubual ini.*
b. *Temubual ini akan mengambil masa kira-kira 10-15 minit. Saya akan bertanya kepada anda beberapa soalan dan anda bebas untuk memberi pandangan berkaitan topik temubual ini. Sesi temubual ini akan dirakam untuk tujuan kajian. Anda bebas untuk menarik diri daripada temubual ini pada bila-bila masa. Ada soalan sebelum kita mulakan?*
c. *Sekarang, saya akan menghidupkan perakam suara.*
d. *Assalamualaikum dan selamat pagi.*
e. *Seperti yang kita tahu, pesakit hemodialisis memerlukan pengurusan pemakanan khusus untuk memastikan kesihatan yang baik. Walau bagaimanapun, isu ekonomi kebelakangan ini mungkin menyebabkan orang ramai tidak mempunyai makanan atau wang yang cukup untuk membeli makanan. Kajian ini berminat untuk mendengar pendapat anda tentang situasi ini.*
   - *Boleh saya tahu, apakah faktor yang menyebabkan anda tidak mempunyai makanan yang cukup atau wang yang mencukupi untuk membeli makanan?*
     (Boleh saya tahu, pada pendapat anda/pakcik/makcik, apa yang menyebabkan sesetengah orang tidak mempunyai makanan yang cukup?
     atau tidak ada wang yang mencukupi untuk membeli makanan
     Prompt: Bagaimana pula keadaan anda/pakcik/makcik?)

- *Berdasarkan pengalaman anda, apa yang anda lakukan apabila tidak mempunyai sumber makanan atau wang yang mencukupi untuk membeli makanan?*

  (Biasanya apa yang orang akan lakukan bila tak cukup makan?

  Atau tak cukup wang untuk membeli makanan?

  Prompt: Bagaimana pula dengan anda/pakcik/makcik? Apa yang anda lakukan?)

f. *Adakah anda mempunyai sebarang komen atau pendapat lain yang ingin dibincangkan mengenai topik yang kita bicarakan pada hari ini?*

g. *Terima kasih atas pendapat yang anda kongsikan pada sesi temubual ini. Semoga informasi yang anda berikan dapat membantu untuk menambah baik kajian ini dan berharap agar kita boleh mencari tahu apa yang boleh dilakukan untuk membantu pesakit-pesakit dialisis yang mengalami kesukaran mendapatkan makanan yang mencukupi. Terima kasih sekali lagi atas penyertaan anda.*
