# Supplementary material for: Food Insecurity Determinants, Coping Strategies, and Association With Nutritional Status Among Hemodialysis Patients in Pahang, Malaysia: Protocol for a Mixed Methods Study
Source: JMIR Res Protoc. 2026 Jan 19;15:e84575. doi: 10.2196/84575 (PMC12904101; doi:10.2196/84575)
Supplement: Multimedia Appendix 2 [file resprot_v15i1e84575_app2.pdf]

MINISTRY OF HIGHER EDUCATION

SINGLE DISCIPLINARY PROJECT

APPLICATION FORM
FUNDAMENTAL RESEARCH GRANT SCHEME (FRGS)

SKIM GERAN PENYELIDIKAN FUNDAMENTAL
(Pindaan 1/2023)

JABATAN PENDIDIKAN TINGGI
KEMENTERIAN PENDIDIKAN TINGGI

Application date	Title	Batch	Approved?	Role	Version	
10/03/2023	(Ref: FRGS/1/2023/SS10/UIAM/02/1)<br>Food insecurity among hemodialysis patients in Pahang: Association with nutritional status, determinants and coping strategies.	FRGS 2023-1	Yes. Approved RM80000<br>Hide	Leader<br>Show members	3<br>Show Older	

Level	Evaluation	Remark
KPT Result	Recommended	Title: title is too long<br>Executive Summary: written in coherent manner<br>Research Background: The operational definition of food insecurity must be reconsidered by taking into consideration the element of wrong selection of food due to "pantang" among the Malays.<br>Objective: objectives are measurable and achievable.<br>Methodology: The methodology is clear and able to achieve the objectives.<br>Expected Result: the result would lead to a new finding, but "Its association with nutritional status" is not explained.<br>Track Record and Composition of Team: a balanced team<br>Quality of Proposal: The proposal is clear and well written.<br>Elements of FRGS Criteria: element of novelty and impactful are there.<br>Research Collaborator: acceptable<br>Patent Search: acceptable<br>Risk Assessment: there is a risk that the number of respondents, if only B40, is inadequate. It would be convenient not to limit to B40 patients only.
